# The Incidence of Paediatric Acute Kidney Injury Identified Using an AKI E-Alert Algorithm in Six English Hospitals

**DOI:** 10.3389/fped.2020.00029

**Published:** 2020-02-11

**Authors:** Sheetal Bhojani, Jelena Stojanovic, Nabil Melhem, Heather Maxwell, Peter Houtman, Angela Hall, Cheentan Singh, Wesley Hayes, Rachel Lennon, Manish D. Sinha, David V. Milford, British Association for Paediatric Nephrology

**Affiliations:** ^1^Royal Hospital for Children, Glasgow, United Kingdom; ^2^Evelina London Children's Hospital, London, United Kingdom; ^3^Leicester Royal Infirmary, Leicester, United Kingdom; ^4^North Middlesex University Hospital NHS Trust, London, United Kingdom; ^5^Bristol Royal Hospital for Children, Bristol, United Kingdom; ^6^Royal Manchester Children's Hospital, Manchester, United Kingdom; ^7^Kings College London, London, United Kingdom; ^8^Birmingham Women's and Children's Hospital, Birmingham, United Kingdom

**Keywords:** acute kidney injury, hospital, epidemiology, alerts, algorithm

## Abstract

**Objective:** Acute kidney injury (AKI) is a significant cause of morbidity and mortality among hospitalised patients. The objectives in this study were (i) to investigate the incidence of AKI using the National Health Services (NHS) AKI e-alert algorithm as a means of identifying AKI; and (ii) in a randomly selected sub-group of children with AKI identified using the algorithm, to evaluate the recognition and management of AKI.

**Patients and Methods:** Retrospective cross-sectional study with initial electronic retrieval of creatinine measurements at six hospitals in England over a six-month period. Results were evaluated using the NHS AKI e-alert algorithm with recognition and management of AKI stages 1, 2 and 3 reviewed in a sub-set of randomly selected patient case notes. Patients aged 29 to 17 years were included. AKI stage 1 was defined as a rise of 1.5 – ≤2x baseline creatinine level; AKI stage 2 a rise of ≤ 2.0 and < 3.0; AKI stage 3 a rise of ≥ 3.0. Urine output was not considered for AKI staging.

**Results:** 57,278 creatinine measurements were analysed. 5,325 (10.8%) AKI alerts were noted in 1,112 patients with AKI 1 (62%), AKI 2 (16%) and AKI 3 (22%). There were 222 (20%) <1y, 432 (39%) 1 ≤ 6y, 192 (17%) 6 ≤ 11y, 207 (19%) 11 ≤ 16y, and 59 (5%) 16–17y. Case notes of 123 of 1,112 [11.1%] children with AKI alerts were reviewed. Confirmed AKI was recognised with a documented management plan following its identification in *n* = 32 [26%] patients only.

**Conclusions:** In this first multicentre study of the incidence of AKI in children admitted to selected hospitals across England, the incidence of AKI was 10.8% with most patients under the age of 6 years and with AKI stage 1. Recognition and management of AKI was seen in just over 25% children. These data highlight the need to improve recognition of AKI in hospitalised children in the UK.

## Introduction

Acute kidney injury (AKI) is characterised by a reversible loss of normal kidney function and is recognised by a reduction in urine output and/or an increase in serum creatinine, indicative of a reduction in glomerular filtration rate (GFR). It is also usually accompanied by an inability of the kidneys to maintain water, acid-base and electrolyte balance (1, 2). The definition of AKI has undergone several iterations (3) and to define and stratify the severity of AKI several classification systems have been proposed and include the RIFLE, AKIN, and KDIGO criteria (4). These consensus definitions are to be welcomed as they help to standardise the diagnosis of AKI, stratify AKI severity and allow the development of predictors of outcomes based on the AKI staging. In children AKI is staged using broadly similar criteria to adults modified as pRIFLE (1).

In adults, AKI is commonly seen in hospitalised patients and associated with increased morbidity and mortality, with worse outcomes in the sickest patients and those with co-morbidities (5–8). Over the past decade several studies have described AKI in hospitalised children and although the incidence is lower, children also have increased morbidity and mortality associated with AKI (9). Recently, Sutherland et al., described rates of AKI in hospitalised children across North America and observed 3.9 episodes of AKI per 1,000 admissions (10). Similar data have been reported from other parts of the world (11, 12).

In those admitted to paediatric ICU, recent data from the AWARE study highlights that AKI is common and is associated with poor outcome including increased mortality (13). Early detection of AKI and implementation of strategies to minimise progression is therefore essential for acute management, but is also likely to improve long term outcomes, particularly in those with the most severe illness (e.g., those admitted to PICU, those undergoing cardiac surgery or those with prematurity), although further data are awaited to prove this (14, 15).

In England, a national algorithm, standardising the definition of AKI was agreed and provides the ability to ensure that a timely and consistent approach to the detection and diagnosis of patients with AKI across the National Health Service (NHS) (16), termed the “NHS AKI e-alert algorithm” in the manuscript [online Supplement]. Following integration of the algorithm into the Laboratory Information Management Systems the algorithm will identify potential cases of AKI from laboratory data in real time and produce a test result. The laboratory system will then send the test result, using existing IT connections to patient management systems in use in hospitals in England. Full implementation across all NHS England sites was intended by 9th March 2015 but has taken longer and this is often due to individual local hospital issues. Nonetheless, studies in adults have shown the implementation of AKI alerts and using an AKI care bundle improved recognition and management of acute kidney injury in hospitalised patients (17, 18).

The objectives of this study were (i) to investigate the incidence of AKI using the NHS AKI e-alert algorithm as a means of identifying AKI; and (ii) in a randomly selected sub-group of children identified using the algorithm, to evaluate the recognition and management of AKI.

## Methods

This project was a retrospective cross-sectional study including six centres across England including three district general hospitals [Leicester Royal Infirmary hospital (Centre #1–centre sees both secondary and tertiary care patients), North Middlesex Hospital (Centre #2) and Worcestershire Acute Hospitals (Centre #3)] and three tertiary children's hospitals [Bristol Royal Hospital for Children (Centre #4), Evelina London Children's Hospital (Centre #5) and Royal Manchester Children's Hospital (Centre #6)]. The participating hospitals in this study are centres geographically placed across England. They are also representative for level of care, including both secondary and tertiary level hospital-based acute services for children in England. The tertiary level participating centres provide specialist paediatric nephrology services including management of children with chronic kidney disease, dialysis, and transplantation [Centres #4, #5, and #6]. This multi-centre project was supported by the membership of the British Association for Paediatric Nephrology and its Acute Kidney Injury Interest Group.

The study was designed in two parts; in the first part of the study, all plasma creatinine measurements performed over a 6 month study period between 01/07/2012 to 31/12/2012 were electronically retrieved and compared to any creatinine measurements that may have been performed over the previous year, allowing staging of AKI using the NHS AKI e-alert algorithm (16). Patients aged 29 days to 16 years 364 days were included in the study. Plasma creatinine was measured using the enzymatic method.

AKI stage 1 was defined as a rise of 1.5 – < 2x baseline creatinine level; AKI stage 2 a rise of ≥ 2.0 and < 3.0; AKI stage 3 a rise of ≥ 3.0. The baseline creatinine was defined as the lowest creatinine value in the previous 12 months (if available) or the upper limit of the reference value for the age (19).

The lead centre (Centre #5) requested electronic retrieval of all creatinine measurements performed at participating centres during the study period. In addition to the plasma creatinine measurements a brief dataset associated with each measurement was included: age, gender, date and time of creatinine measurement, stage of AKI and number of AKI alerts. All centres sent data electronically to the lead centre and data analysed by investigators.

In the second part of the study, a subset of children identified to have AKI were randomly selected for case note review from 5 of 6 centres. Study investigators visited participating centres to collect data from paper and electronic case notes. Notes from Centre #2 were unavailable for review.

Data collected included patient demographics, documented evidence of AKI recognition by the clinical team, the stage of AKI, if appropriate investigations and management were carried out, and if a specialist nephrology opinion was sought and outpatient follow up arranged. This was a retrospective analysis evaluating the incidence of AKI from results of clinically indicated investigations and therefore no consent from patients and ethical approval was required. There was no direct patient or public involvement in this study.

## Statistical Analyses

Summary statistics are presented as means for continuous data and median and inter-quartile range (IQR) for non-normally distributed data. Comparisons between the two groups in this study were tested as appropriate using the independent samples *t*-test for continuous data, Mann–Whitney *U*-test for non-normally distributed data and the Chi–squared test for categorical data. Statistical analyses were performed using SPSS version 22 (SPSS Inc., Chicago, Illinois). All tests were two-tailed and a *p* < 0.05 was taken to represent a statistically significant result.

## Results

**Figure 1:**
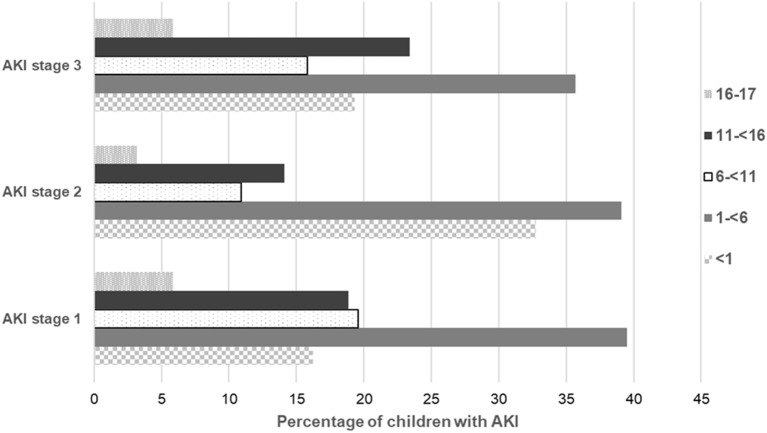
Distribution of *n* = 1,112 children with AKI alerts shown as percentage of children in each age band by worst stage of AKI. Data by worst stage of AKI as indicated by maximal change in creatinine level from baseline using the National Health Service (NHS) AKI e-alert algorithm (16).

**Figure 2:**
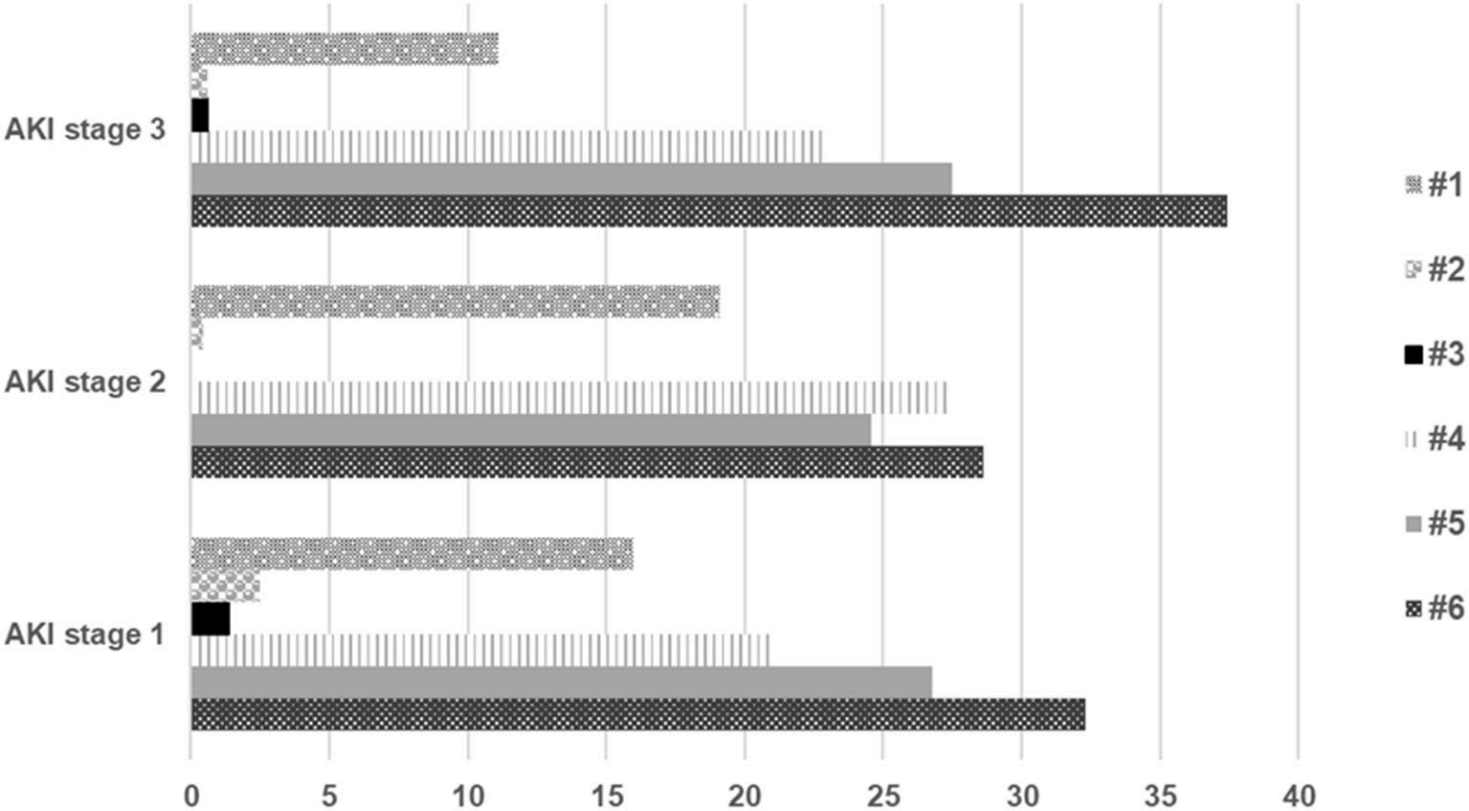
Incidence of AKI alerts in *n* = 1,112 patients shown by percentage of children in each centre* by worst stage of AKI. Data by worst stage of AKI as indicated by maximal change in creatinine level from baseline using the National Health Service (NHS) AKI e-alert algorithm (16). *Centre #1, Leicester Royal Infirmary hospital; Centre #2, North Middlesex Hospital; Centre #3, Worcestershire Acute Hospitals; Centre #4, Bristol Royal Hospital for Children; Centre #5, Evelina London Children's Hospital; Centre #6, Royal Manchester Children's Hospital.

57278 creatinine measurements over a 6 month period were retrieved from the six centres; measurements from individual centres are shown in Table 1. Of these 75.2% measurements [*n* = 43,080] had a baseline creatinine measurement over the previous year to compare with and 24.8% [*n* = 14,198] creatinine measurements had no previous baseline measurements. There were 5,325 [10.8%] creatinine values resulting an AKI alert in 1,112 patients using the NHS AKI e-alert algorithm. The incidence of AKI was higher when restricted to those in whom a previous creatinine measurement 12.4% [5325/43080] was available. Of these AKI alerts, 33,24 (62%) were AKI stage 1, 843 (16%) were AKI stage 2 and 1,158 (22%) were AKI stage 3 alerts. In those with no previous baseline measurement, 7.7% [1088/14198] had creatinine measurement above the upper limit of the reference value for their age and this was significantly lower than those in whom a baseline creatinine was available and who had an AKI alert [*p* < 0.0001]. The age distribution of children with AKI alerts was 222 (20%) <1 year, 432 (39%) 1 ≤ 6 years, 192 (17%) 6 ≤ 11 years, 207 (19%) 11 ≤ 16 years, and 59 (5%) 16–17 years. Their distribution by percentage in each age band by worst stage of AKI is shown in Figure 1. AKI stage 1 was the largest group across all ages and 59% of all AKI alerts occurred in those aged <6 years. In those patients with AKI, there were 176, 20, 11, 251, 294, and 360 children with AKI in Centres #1, #2, #3, #4, #5, and #6, respectively, and a statistically significant difference in the incidence of AKI between the centres [*p* < 0.0001]. The incidence of AKI in 1,112 patients shown by percentage of children in each centre by worst stage of AKI is shown in Figure 2.

**Table 1:** Number of creatinine measurements per centre analysed over a 6 month period across six participating centres^*^, including *n* = 57,278 measurements.

**Centre**	**Number of measurements**
#1	14,029 (24.5%)
#2	1,775 (3.1%)
#3	965 (1.7%)
#4	7,900 (13.8%)
#5	12,095 (21.1%)
#6	20,514 (35.8%)

* *Centre #1, Leicester Royal Infirmary hospital; Centre #2, North Middlesex Hospital; Centre #3, Worcestershire Acute Hospitals; Centre #4, Bristol Royal Hospital for Children; Centre #5, Evelina London Children's Hospital; Centre #6, Royal Manchester Children's Hospital*.

In the second part of the study, 123 of 1,112 (11.1%) patients with AKI alerts were randomly selected for case notes review across 5 of the 6 centres (Table 2). There were 68 males (55.2%). The median [IQR] age was 3.1 years (0.5, 6.6). AKI was correctly identified and a management plan following its identification was documented in 32 (26%) of patients. In 87 (71%) patients, AKI was not identified with no clinical notes highlighting this issue or no management plan for AKI; and for 4 (3%) patients there was no information available.

**Table 2:** Clinical characteristics of randomly selected patients with AKI from five centres^*^ identified retrospectively using National Health Service (NHS) AKI e-alert algorithm.

**Centres**	**1**	**3**	**4**	**5**	**6**
Number of patients, *n* (%)	29 (23.5%)	11 (8.9%)	28 (22.8%)	25 (20.3%)	30 (24.3%)
Age in years [median (IQR)], *n* = 95	2.0 (0.5, 3.3)	7.5 (0.6, 12.7)	No data available	2.3 (0.4, 6.6)	3.9 (1.8, 6.4)
Male, *n* (%)	15 (51.8%)	6 (54.6%)	14 (50%)	16 (64%)	17 (56.7%)
Creatinine in μmol/L at presentation [median (IQR)]	45 (36, 61)	46 (36, 85)	71 (51, 141)	44 (33, 86)	27 (21, 46)
Correctly identifed AKI, *n* (%)	3 (10.3%)	1 (9%)	11 (39%)	11 (44%)	6 (20%)
Management plan accounting for AKI					
Yes	4 (13.7%)	2 (18%)	13 (46.4%)	7 (28%)	6 (20%)
No	24	8	13	18	24
No information	1	1	2	0	0

* *Centre #1, Leicester Royal Infirmary hospital; Centre #3, Worcestershire Acute Hospitals; Centre #4, Bristol Royal Hospital for Children; Centre #5, Evelina London Children's Hospital; Centre #6, Royal Manchester Children's Hospital*.

Data for patients in whom AKI was recognised (*n* = 32; AKI-R) was compared to those in whom AKI was not recognised (*n* = 87; AKI-NR) and their management plan following recognition reviewed (Table 3).

**Table 3:** Results of case note review for 119 of 123 patients for clinical management of recognised vs. unrecognised AKI during inpatient episode.

**Demographics**	**AKI-Recognised**	**AKI-Not recognised**
Number of patients (*n* = 123)^∧^, *n* (%)	32 (26%)	87 (71%)
Age in years [median (IQR)]	3.1 (0.7,6.4)	3.1 (0.5,7.2)
Boys, *n* (%)	19 (59.3%)	49 (53.8%)
Creatinine in μmol/L at presentation [median (IQR)]^∧∧^	103 (61,239)	39 (29,57)
**Patients admitted under**		
Total	32	87
General Paediatrics	3	29
Cardiology	2	17
Neurology	0	2
Nephrology	13	1
Neonatology	2	1
Intensive care and ECMO	6	8
General surgery	0	2
Oncology	0	6
Specialty not known	6	21
**Management**		
Daily weight measurements *n* (%)	22 (69%)	13 (14%)
Twice daily weights, *n* (%)	4 (12%)	2 (2%)
Blood pressure measured, *n* (%)	31 (97%)	62 (68%)
Fluid balance, *n* (%)	29 (91%)	39 (43%)
Repeat creatinine measured, *n* (%)	31 (97%)	57 (63%)
Urine dipstick performed, *n* (%)	16 (50%)	13 (14%)
Renal Ultrasound scan performed, *n* (%)	15 (47%)	4 (4%)
Estimated GFR measured, *n* (%)	9 (28%)	1 (1%)
Role of drug related nephrotoxicity and AKI considered, *n* (%)	19 (59%)	1 (1%)
Paediatric Nephrology advice considered, *n* (%)	12 (37%)	1 (1%)
**Follow up**		
^*^OPD clinic review, *n* (%)	28 (90%)	50 (55%)
^**^GP review, *n* (%)	0	1 (1%)
**Creatinine returned to baseline levels**, ***n*** **(%)**		
Yes	25 (78%)	49 (54%)
No	5 (16%)	11 (12%)
Not re-measured	0	18 (20%)
No information	1 (3%)	13 (14%)
Died	1 (3%)	0

*All patients identified retrospectively using National Health Service (NHS) AKI e-alert algorithm*.

∧ *no, information available for 4 patients*;

∧∧ *P < 0.0001*;

* *OPD, outpatient department*;

** *GP, general practitioner*.

Median [IQR] age in years was the same in AKI-R and AKI-NR [3.1 (0.7, 6.4) vs. 3.1 (0.5,7.2), *p* = 0.56]. Boys were affected more in both groups AKI-R (59.3%) vs. AKI-NR (53.8%). Median [IQR] creatinine (μmol/L) was significantly higher in the AKI-R group compared to the AKI-NR group [103 (61,239) vs. 39 (29,57), *p* < 0.0001]. The largest number of AKI cases that were not recognised were admitted in General Paediatrics, followed by Cardiology, Intensive Care, and Oncology.

In terms of management of AKI, the risk of possible drug nephrotoxicity was considered in 59% in AKI-R group compared to 1% in AKI-NR group. A specialist nephrology opinion was sought in 37% vs. 1% in the AKI-R vs. AKI-NR group. 90% of the patients in AKI-R group were followed up in clinic compared to 55% in AKI-NR group (Table 3). Estimated glomerular filtration rate (eGFR) was rarely measured by non-renal specialties. At the time of discharge, serum creatinine had returned to baseline levels in 78% of patients. There was 1 death (3%) in the AKI- R group and none in the AKI-NR group.

## Discussion

This is the first multicentre study looking at the incidence and management of AKI in hospitalised children in selected English hospitals using a creatinine-based algorithm and includes a large data set of children over a 6 month period from both district general and teaching hospitals.

The number of studies of AKI in paediatric population are much less than in the adult population (20) and usually include small numbers of patients. Most of the paediatric studies are either single centre or, if multicentre, focused on high risk groups such as children in PICU whereas this study looked at the incidence of AKI among all hospitalised patients.

In this study here, the overall incidence of AKI was 10.8% which was similar to other studies (12, 21). This was less than the incidence reported in meta-analysis including studies across the world, that reported a pooled incidence of AKI in children of 33.7% (20). However, on inspecting the paediatric studies in the meta-analysis, 67% of the studies included patients in critical care or post cardiac surgery, and study numbers were generally small, therefore their results cannot be considered to represent the true incidence of AKI in all childhood admissions worldwide.

AKI stage 1 was the commonest stage across all ages in this study. The incidence of AKI was higher in the younger age group (~60% of AKI was in children under 6 years of age) which is similar to other studies (11, 21) but contrary to a large epidemiological study by Sutherland et al. where the highest incidence of AKI was seen in 15–18 year olds (10). However, Sutherland et al. used ICD-9-CM codes (International Classification of Diseases, Ninth Revision, Clinical Modification) applied at discharge rather than the creatinine-based criteria of AKIN or pRIFLE criteria for diagnosing AKI (10). Younger children tend to have lower serum creatinine values and therefore a small absolute change in the creatinine value could lead to identification of AKI using the AKIN or pRIFLE criteria but not with the ICD-9-CM coding system (10).

In this study reported here, the pRIFLE or AKIN criteria were not used by clinicians for routinely diagnosing AKI and so patients with a rise in creatinine from baseline but whose creatinine was still within the laboratory specified normal range were less likely to be recognised to have AKI. This includes the group of children who would be at risk of AKI (pRIFLE “R”) and in whom appropriate intervention could be anticipated to prevent progression. It is important to note that in the sub-set of randomly selected patients who had case note review, 74% of patients with AKI were not recognised. Even in the group of children where AKI had been recognised (AKI-R), appropriate management was not implemented, highlighting the need for education and training among the medical staff.

The diagnosis of AKI is usually made by identifying increased serum creatinine with or without associated reduced urine output (17). Studies have shown that using a care bundle can improve the recognition of AKI which can lead to early initiation of treatment to reduce progression of AKI and so reduce morbidity and mortality (17, 18). The AKI alert algorithm was introduced across NHS England in 2015 to facilitate early diagnosis. In this study the data set was from a period before the introduction of the algorithm; further studies will be needed to examine if the early identification of AKI as a result of implementation of the NHS algorithm has led to improved recognition and management of AKI with a reduction in AKI related morbidity and mortality.

There are some important limitations to our study findings that need to be considered and include (i) Inherent biases related to the retrospective nature of such data; (ii) electronic alerts of AKI were based on serum creatinine values only and do not have any information regarding GFR or urine output. We recognise that in clinical practice and in widely established AKI criteria, urine output is integrated to facilitate early diagnosis and lack of these data are therefore likely to underestimate AKI, however we wished to examine the application of the NHS AKI e-alert algorithm in routine clinical practice; (iii) Although the algorithm is helpful for identifying AKI, we recognise in clinical practice use of creatinine alone may be misleading particularly in younger children who have physiologically low creatinine values; similar issues are likely to complicate evaluation in children with chronic illness and malnutrition; (iv) only one definition of AKI stage 3 was used. We recognise that sometimes an increase in serum creatinine may be <3× the baseline but the eGFR for the patient much worse and clinically significant; (v) In those patients in whom no creatinine values were available to compare an increase from a previous baseline, we used the upper limit of reference range as baseline creatinine. In those with a single high value we have not reported this as AKI here as per the algorithm and this may underestimate the true incidence of AKI in our cohort. Previous studies (22–24), have shown increased sensitivity in detecting AKI when using the lower reference limit or using back calculation to estimate the baseline creatinine from the Schwartz formula and defining baseline GFR as 120 ml/min/1.73m^2^. Importantly, these approaches may categorise those with CKD as AKI but also overestimate the true incidence of AKI; furthermore back calculation requires the patient height. Despite this as shown in this study, there remain significant shortcomings in the recognition of AKI by non-renal physicians, with poor recording of urine output and/or of eGFR for hospitalised children receiving clinical care. A creatinine based algorithm embedded in hospital reporting systems is likely to be more effective in identifying AKI.

## Conclusion

The incidence of AKI in this multicentre study was 10.8% with most patients under the age of 6 years and AKI stage 1 was the predominant stage across all ages. There remains an ongoing need for education and training of health professionals to improve recognition and management of patients with AKI. Timely recognition and optimal management of AKI is important to improve long term renal outcomes. Future studies will aim to determine the impact of the NHS AKI e-alert algorithm in the UK.

## Data Availability Statement

All relevant data is contained within the article.

## Ethics Statement

The study was registered with the corresponding authors institution as a multi-centre audit. This was a review of historical hospital results/records. No ethical approval was required following audit approval. Written informed consent from the participants' legal guardian/next of kin was not required to participate in this study in accordance with the national legislation and the institutional requirements.

## Author Contributions

SB: data collection, manuscript writing, final approval of the version to be published, and agreement to be accountable for all aspects of the work in ensuring that questions related to the accuracy or integrity of any part of the work are appropriately investigated and resolved. MS and DM: project leads, designing the project, data collection, analysis, manuscript writing, final approval of the version to be published, and agreement to be accountable for all aspects of the work in ensuring that questions related to the accuracy or integrity of any part of the work are appropriately investigated and resolved. JS, NM, HM, PH, AH, CS, WH, and RL: data collection from individual centres and approval of the final version.

### Conflict of Interest

The authors declare that the research was conducted in the absence of any commercial or financial relationships that could be construed as a potential conflict of interest.
