# Supplementary material for: The Incidence of Paediatric Acute Kidney Injury Identified Using an AKI E-Alert Algorithm in Six English Hospitals
Source: Front Pediatr. 2020 Feb 11;8:29. doi: 10.3389/fped.2020.00029 (PMC7026188; doi:10.3389/fped.2020.00029)
Supplement: Supplementary file 1 [file Data_Sheet_1.pdf]

Algorithm for detecting Acute Kidney Injury (AKI) based on serum creatinine changes with time

This algorithm relates to the NHS England patient safety alert: NHS/PSA/D/2014/010

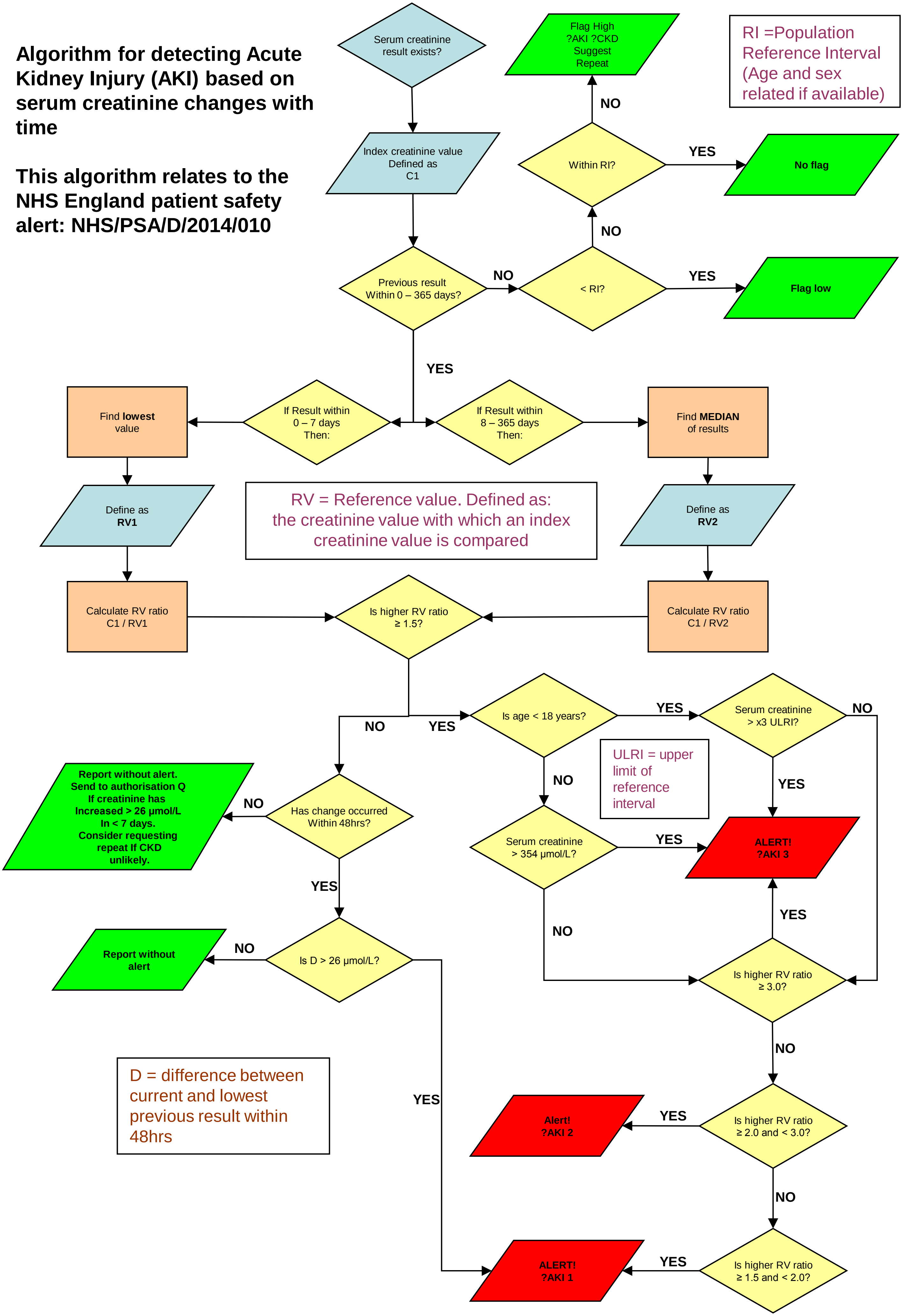

Acknowledgements

Algorithm for detecting Acute Kidney Injury (AKI) based on serum creatinine changes with time

This algorithm relates to the NHS England patient safety alert: NHS/PSA/D/2014/010

Conceptual design: Members of the consensus group whose names and meeting report can be accessed on:

http://www.acb.org.uk/docs/E-Alerts_for_AKI_meeting_statement

Graphic design: Robert Desborough, Mike Bosomworth, Robert Hill
